# Role of RAC3 coactivator in the adipocyte differentiation

**DOI:** 10.1038/s41420-018-0085-y

**Published:** 2018-07-26

**Authors:** María Cecilia Lira, Francisco Damian Rosa, Laura Carolina Panelo, Mónica Alejandra Costas, María Fernanda Rubio

**Affiliations:** 1 Universidad de Buenos Aires, Facultad de Medicina, Instituto de Investigaciones Médicas A Lanari, Buenos Aires, Argentina; 2 Consejo Nacional de Investigaciones Científicas y Técnicas, Laboratory of Molecular Biology and Apoptosis, Instituto de Investigaciones Medicas (IDIM), Universidad de Buenos Aires, Combatientes de Malvinas Av 3150, CABA, Argentina, Buenos Aires, Argentina

**Keywords:** Molecular biology, Autophagy

## Abstract

RAC3 is a member of the p160 family of steroid receptor coactivators and it is highly expressed in several human cancers, contributing to enhanced cell proliferation and cellular transformation. In this work, we have studied the role of RAC3 in adipogenesis in L-929 cells. Adipogenesis is a highly regulated process, involving cell cycle arrest and changes in the gene expression pattern required for morphological remodelling. We found that RAC3 expression levels are downregulated during adipocyte differentiation induced by specific stimulus. In addition, cells constitutively expressing low levels of RAC3 (shRNA), showed enhanced adipocyte differentiation which was evidenced by the early detection of the adipocyte markers Perilipin, PPARγ and Oil Red O staining. Moreover, RAC3 downregulation favoured cell arrest and autophagy. Early and late autophagy inhibitors blocked adipocyte differentiation in control cells, but partially inhibited shRAC3 differentiation, demonstrating that although autophagy is required for adipogenesis, additional signals could be trigged by RAC3 downregulation. We conclude that RAC3 is a key regulator of adipogenesis, since its downregulation generates the cellular arrest and autophagic responses that are required steps for this process.

## Introduction

The adipose tissue is formed by cells with lipid depots called adipocytes. The perception of this tissue only as a storage place has been replaced in recent years by the notion that adipocytes have a central role in lipid and glucose metabolism and produce a large number of hormones and cytokines, called adipokines^1,2^. These molecules participate in complex endocrine, paracrine and autocrine signalling networks^2^.

Adipokines secreted from adipose tissue have been recognized for their contribution to the mechanisms by which obesity and related metabolic disorders increase cancer risk^3^. Obesity induces a chronic inflammatory state in which adipose tissue cells secrete an increased amount of adipokines^4^. Not only are adipocytes affected by this state, but also fibroblasts that have the potential to differentiate into mature adipocytes, and macrophages that infiltrate the mass of adipose cells^5^.

Given the close relationship between adipose tissue and cancer development, it is important to improve the knowledge concerning the stimuli and mechanisms that give rise to adipogenesis in order to better understand its role in the risk of cancer development^6^.

Adipocyte differentiation is a multi-step process, involving activation of transcription factors and cofactors recruitment to promoter sequences of target genes required for terminal differentiation^7^. In particular, the transcription factors C/EBPs (β, δ and α) and PPARγ are required for adipogenesis. Thus, the action of PPARγ and C/EBP-α lead to the expression of genes that is necessary to maintain adipocyte phenotype^8,9^. Different coactivators have been reported to favour the action of these transcription factors, among them PGC-1α, and members of the p160 family of steroid receptor coactivators (SRCs)^10^.

RAC3 (Receptor Associated-Coactivator3) is a member of the p160 family of SRCs. In particular, this coactivator is highly expressed in several human cancers^11–13^. However, in recent years, the physiological role of RAC3 has been investigated^14^ and it has been demonstrated that its expression is required for the maintenance of pluripotency^15^.

We have previously found that RAC3 overexpression has an anti-apoptotic, anti-autophagic and pro-proliferative role^16–19^. In this study we demonstrate that RAC3 expression is a key regulator of adipogenesis and its downregulation accelerates this process, enhancing cellular arrest and autophagic responses.

## Results

### The induction differentiation medium triggers adipogenesis in murine fibroblastic L-929 cell line

**Fig. 1:**
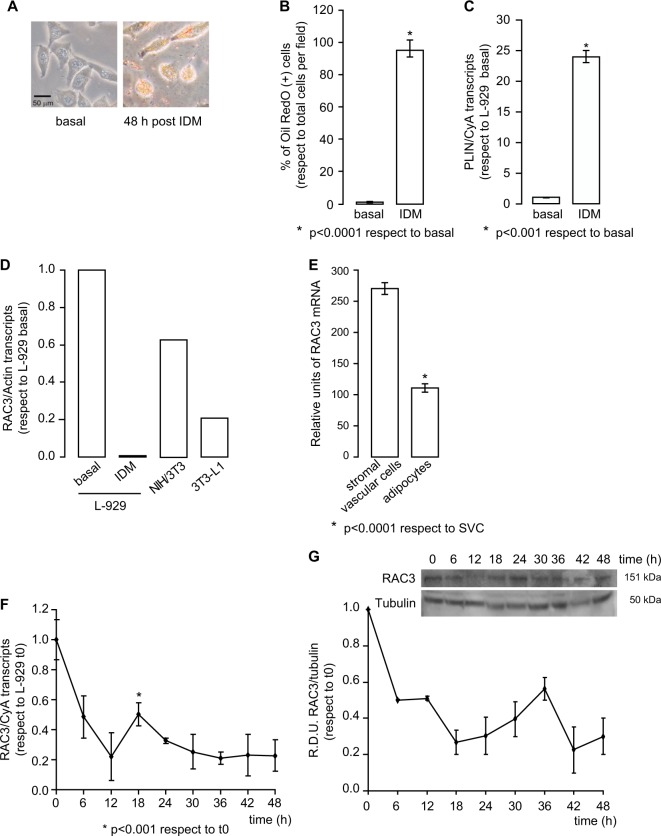
IDM induces L-929 adipocyte differentiation. **a** Representative images of the L-929 cell line stimulated or not with IDM for 48 h. Cells were stained with Oil Red O. **b** Diagram bars correspond to percentage of Oil Red O positive cells per field (at least 10 fields per sample). Student test was performed (*n* = 3), *p < 0.0001 with respect to basal condition. **c** Perilipin (PLIN) mRNA expression in basal condition or 48 h post induction with IDM was determined by qPCR and normalized with Cyclophilin A (CyA) mRNA. Student test was performed (*n* = 3), *p < 0.001 with respect to basal condition. **d** RAC3 expression levels were evaluated by qPCR in L-929 cells in basal conditions or 48 h post IDM treatment, NIH/3T3 and 3T3-L1 cells, and were normalized to Actin mRNA. **e** RAC3 expression levels of stromal vascular cells (SVC) and adipocytes from murine epididymal tissue were compared. The diagram bars show the average ± SD of mRNA expression log-transformed values from GSE65557 data bank *p < 0.0001 with respect to SVC. **f**, **g** Temporal RAC3 expression levels, after IDM treatment, were analysed by **f** qPCR or **g** western blot. **f** Each point corresponds to average ± SD of RAC3 mRNA expression obtained by qPCR and normalized to CyA mRNA, *p < 0.001 with respect to t0. **g** Western blot was performed to determine RAC3 protein levels, relative densitometry units (RDU) correspond to the densitometry unit with respect to Tubulin expression. Inset corresponds to representative immunoblot

Since not all fibroblasts are able to differentiate into the same lineages^20^, we first investigated the capacity of the murine fibroblastic cell line L-929 to differentiate to adipocytes by using the typical induction differentiation medium (IDM)^21^. We observed that treatment for 48hs with IDM increases the percentage of positive cells for Oil Red O staining (Fig. 1a, b). We found that cells treated with IDM had a large number of lipid depots and showed a typical morphology of adipocytes, characterized by rounded cells. In contrast, no vesicles were observed in basal conditions (Fig. 1a).

Perilipin (PLIN) is a protein present in lipid vesicle membrane and it is considered a marker of adipocyte phenotype. We observed upregulation of PLIN mRNA expression levels after 48hs of IDM treatment (Fig. 1c).

Once the ability of L-929 cell line to differentiate into adipocytes had been determined, RAC3 expression levels were analysed in L-929 cells at basal condition or after 48 h post treatment with IDM, NIH/3T3 or 3T3-L1. We found that the differentiated cells expressed lower levels of RAC3 than the parental cell line (Fig. 1d). These results were similar to those obtained by bioinformatic analysis of stromal vascular cells (SVC) and adipocytes cells extracted from murine epididymal adipose tissue (Fig. 1e)^22^.

Next, we analysed if RAC3 expression could be modulated in the course of adipocyte differentiation. We found that its expression levels were downregulated during the differentiation process (Fig. 1f, g). Concerning the kinetics of RAC3 modulation, a slow increase in RAC3 mRNA expression was observed at 18 h post induction, though it remained below the basal levels in control cells (Fig. 1f). Moreover, RAC3 protein levels remained low at all times studied (Fig. 1g).

Therefore, L-929 cells are able to differentiate into adipocytes using IDM and this process involves the downregulation of RAC3.

### Constitutive low RAC3 expression accelerates the differentiation to adipocytes

**Fig. 2:**
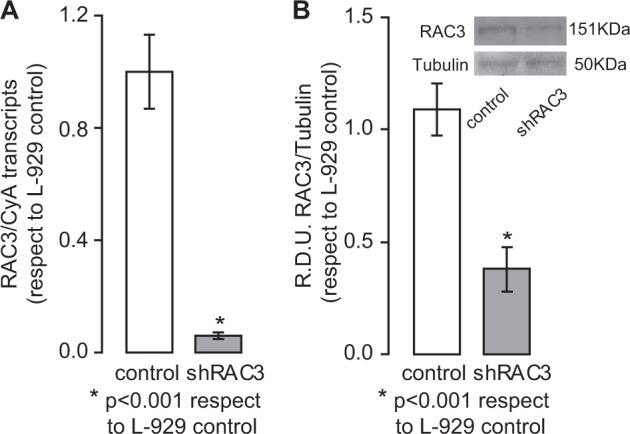
Validation of the decrease in RAC3 expression levels. **a**, **b** Analysis were performed to validate knockdown efficiency of shRAC3. **a** RAC3 mRNA expression was determined by qPCR and was normalized to CyA mRNA expression. Student test (*n* = 3), *p < 0.001 with respect to control L-929 cells. **b** Anti-RAC3 western blot analysis; RDU correspond to the average of densitometry unit with respect to Tubulin expression. The diagram bars correspond to RDU average ± SD of five independent experiments from protein extracts of cells at different post-selection passage. Inset: representative immunoblot. Student test (*n* = 5), *p < 0.001 with respect to control cells

To explore the potential role of RAC3 in adipocyte differentiation, the L-929 cell line was transfected with a plasmid containing the shRNA sequence for murine RAC3 (shRAC3) or a scramble sequence (control). Western blot and qPCR analysis were performed to validate the knockdown efficiency (Fig. 2a, b).

**Fig. 3:**
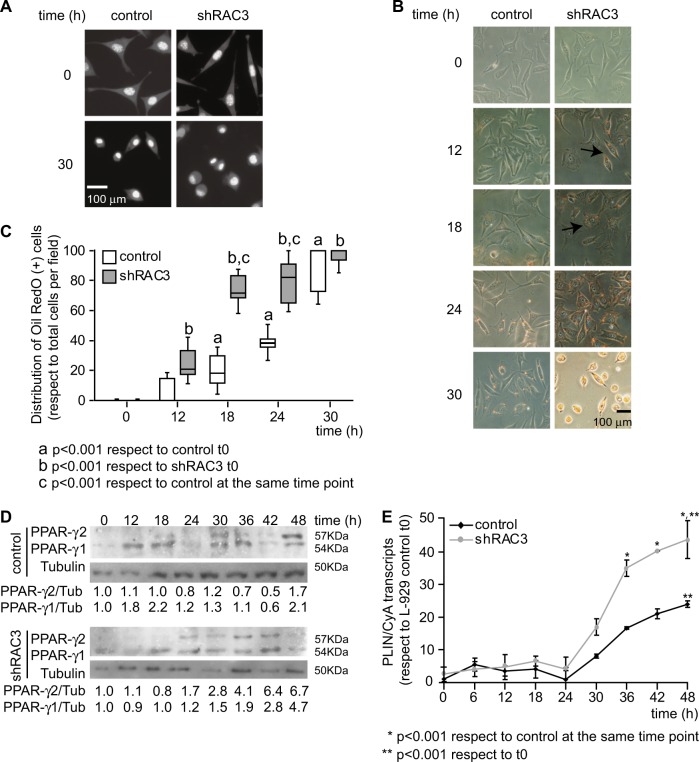
The decrease of RAC3 expression accelerates the expression of adipocyte molecular markers. **a** Representative photographs of Hoechst 33342 staining in basal condition or at 30 h post induction with IDM. **b** Light microscopy photographs of control and shRAC3 L-929 cell lines in basal conditions or stimulated with IDM at different times, were stained with Oil Red O. The arrow shows positive Oil Red O staining of lipid droplets. **c** Diagram bars correspond to percentage of Oil Red O positive cells per field (at least 10 fields per sample). Statistical analysis ANOVA and Tukey post-test of three independent experiments were performed, a and b *p* < 0.001 with respect to control and shRAC3 cells in basal conditions, respectively; and c *p* < 0.001 with respect to control cells at the same time point. **d** PPARγ 1/2 levels were evaluated by western blot. RDU correspond to densitometry unit with respect to Tubulin expression. **e** Temporal modulation of PLIN expression in control and shRAC3 L-929 cells after adipocyte differentiation. ANOVA (*n* = 3) *p < 0.001 with respect to control at the same time point and ** *p* < 0.001 with respect to t0

Fluorescence photographs showed a morphological change in cells stimulated with IDM, characterized by rounded cells and eccentric nuclei with condensed DNA, consistent with typical adipocyte morphology (Fig. 3a). The analysis of molecular markers of adipocyte differentiation, like PPARγ 1/2, PLIN and Oil Red O staining, showed that the decrease in RAC3 expression accelerates the appearance of these markers after IDM treatment in both cell lines. The presence of lipid vesicles was evaluated by Oil Red O staining at different time points after IDM stimulation. Cells positive for this staining were observed earlier in shRAC3 L-929 than in control cells. Although at 24 h after induction, almost 100% of the shRAC3 cells were positive for this dye, in control cells this effect was only observed after 30 h (Fig. 3b, c).

PPARγ 2 is a characteristic marker of adipose tissue^9^ and we found it was expressed earlier in shRAC3 L-929 cells when compared to the control cell line. A significant increase of PPARγ 2 expression was observed at 24 h in shRAC3 cells while in the control cell line this increase was observed after 48 h of IDM treatment (Fig. 3d). When we assessed PLIN expression, the IDM treatment for 30 h increased mRNA expression levels in control cells, but again, it was significantly higher in cells expressing low levels of RAC3 (Fig. 3e).

Altogether, these results indicate that the role of RAC3 downregulation is to accelerate adipogenesis.

### RAC3 downregulation induces cell cycle arrest

**Fig. 4:**
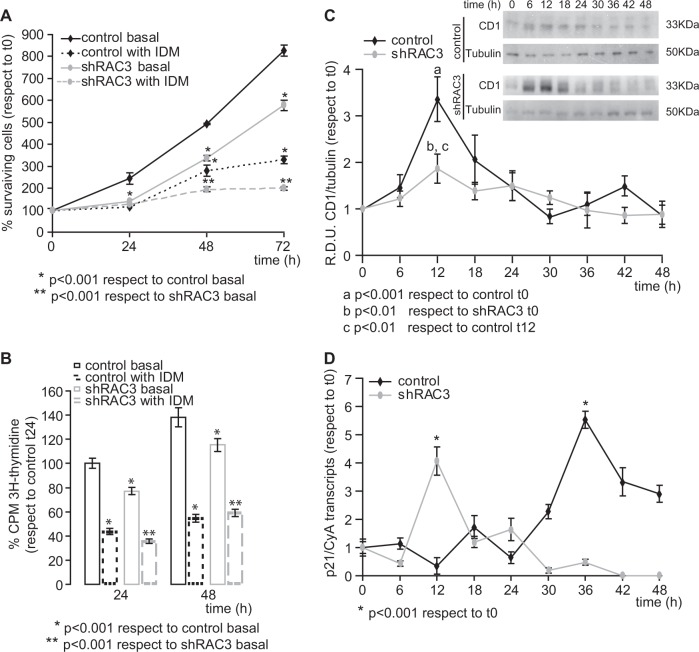
RAC3 downregulation is required for induction of cell arrest. **a**, **b** Cell viability was determined by **a** crystal violet staining or **b** tritiated thymidine uptake assays in control and shRAC3 L-929 cells treated or not with IDM at different times. Curves and diagram bars represent the average ± SD percentage of surviving cells with respect to 0 h (*n* = 3), * and **p < 0.001 with respect to control and shRAC3 L-929 cells in basal conditions, respectively. **c**, **d** Temporal modulation of Cyclin D1 (CD1) (**c**) and p21 (**d**) expression levels in control and shRAC3 L-929 cells after adipocyte differentiation induction. **c** CD1 protein levels were evaluated by western blot at different time points after IDM stimulation, curves represent RDU corresponding to the average of densitometry units with respect to Tubulin expression, (*n* = 3) a *p* < 0.001 with respect to control L-929 cells in basal conditions, b *p* < 0.01 with respect to shRAC3 L-929 cells in basal conditions and c *p* < 0.01 with respect to control L-929 cells at 12 h post induction. Inset corresponds to representative immunoblot. **d** p21 mRNA was evaluated by qPCR and normalized to CyA mRNA expression. Curves represent the average ± SD of three independent experiments, *p < 0.001 with respect to basal condition

Cellular arrest is a required step for cell differentiation^23^. In addition, it is known that expression levels of RAC3 are regulated during the cell cycle^24,25^. Therefore, cell viability was tested by crystal violet staining (Fig. 4a) and tritiated thymidine uptake assays (Fig. 4b). By using both techniques, we found that in the absence of IDM, shRAC3 cells showed lower proliferation than control cells (Fig. 4a, b). These results are consistent with our previous findings demonstrating that RAC3 expression is necessary for cell proliferation^25^. On the other hand, although IDM treatment induced a decrease in the proliferation rate in both cell lines as expected, this effect was stronger in shRAC3 cells (Fig. 4a, b).

In order to evaluate whether the decrease in the proliferation rate could be mainly a consequence of the cell cycle arrest, we analysed the expression levels of Cyclin D1 (CD1) and p21, involved in cell cycle regulation and G1 arrest, respectively.

We found that CD1 protein expression showed a peak at 12 h post induction in both cell lines, nevertheless, this increase was lower in shRAC3 cells (Fig. 4c). Interestingly, in control cells after 30 h post treatment, CD1 expression fell below the levels obtained in basal condition. This result was observed when the maximal expression of adipocyte phenotypic markers was obtained. Concerning p21 mRNA expression, we observed an increase at 12 h post IDM treatment in shRAC3 cells, while in the control L-929 cells this peak was found at 36 h post stimulation (Fig. 4d).

Therefore, IDM induces cell arrest in L-929 cell line and this effect is enhanced by the decrease in RAC3 expression levels.

### The IDM stimulation favours autophagy

Autophagy is involved in different physiological and pathological processes^26,27^. In particular, during adipocyte differentiation, cells need to recycle cellular components using autophagy to restructure the cytoplasm^28^. On the other hand, we have previously demonstrated that RAC3 overexpression inhibits autophagy^18^.

**Fig. 5:**
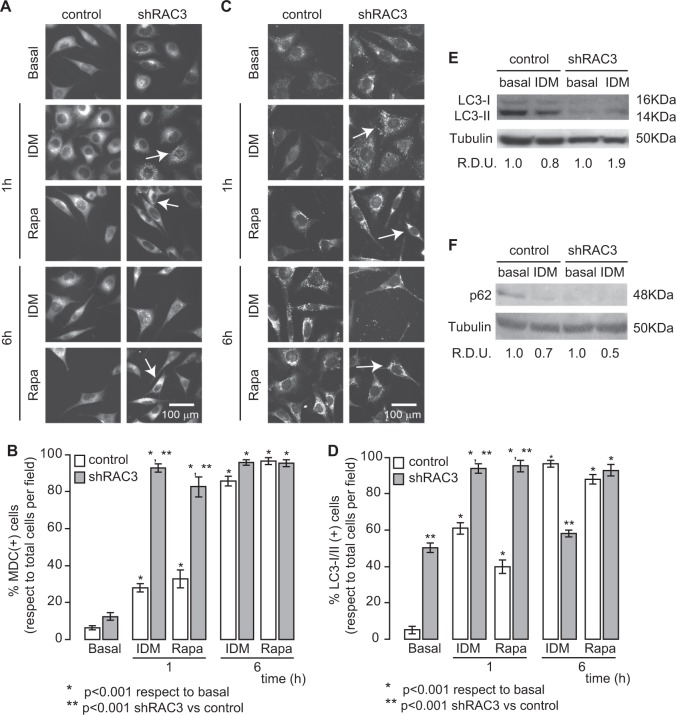
IDM induces autophagy. **a**–**d** Control and shRAC3 L-929 cells were seeded in 24-well plates with slices and, after 24 h, cells were stimulated with IDM or Rapamycin (Rapa 0.5 μM). **a**, **b** Autophagy was evaluated by staining with monodansylcadaverine (MDC) or **c**, **d** immunofluorescence using an anti-LC3-I/II antibody and an anti-rabbit coupled to FITC antibody after 1 or 6 h post treatment. The arrows show MDC or LC3-I/II positive vesicles, respectively. **b**, **d** Diagram bars correspond to percentage of MDC or LC3-I/II positive cells per field (at least 10 fields per sample). Statistical analysis ANOVA and Tukey post-test *n* = 3 were performed, *p < 0.001 with respect to basal condition, **p < 0.001 with respect to control L-929 cells. **e**, **f** LC3-I/II (**e**) and p62 (**f**) levels were determined by western blot after 90 min post IDM treatment. RDU correspond to the average of densitometry unit with respect to Tubulin expression

Thus, to study the role of autophagy during adipocyte differentiation, control or shRAC3 L-929 cell lines were stimulated with the autophagy inductor Rapamycin (Rapa 0.5 μM) or IDM. By both monodancyl cadaverine (MDC) staining of acidic vesicles (Fig. 5a, b) and LC3-I/II immunofluorescence (Fig. 5c, d), we observed that IDM induced autophagy in the same proportion as Rapa. Autophagy was significantly higher in shRAC3 L-929 cells in all experimental conditions. In particular, this cell line showed a high percentage of positive cells for LC3-II in basal conditions (Fig. 5d). This result mimics that obtained in human cell lines when RAC3 expression levels are decreased^18^. Furthermore, the increase in LC3-II levels was observed in shRAC3 cells according to the western blot analysis (Fig. 5e).

Interestingly, in shRAC3 cells at 6 h post IDM induction, a decrease in LC3-II labelling was observed, indicating that the process was switched off in order to avoid an excess in autophagy that could trigger apoptosis^29^.

By western blot analysis of p62, we found that IDM induced autophagic degradation of p62 and this effect was potentiated in shRAC3 cells (Fig. 5f).

In fact, we demonstrated for the first time that IDM is able to induce autophagy in the L-929 cell line and this process may be potentiated in cells expressing low levels of RAC3.

### The decrease in RAC3 expression levels favours adipogenesis through an increase in autophagy

**Fig. 6:**
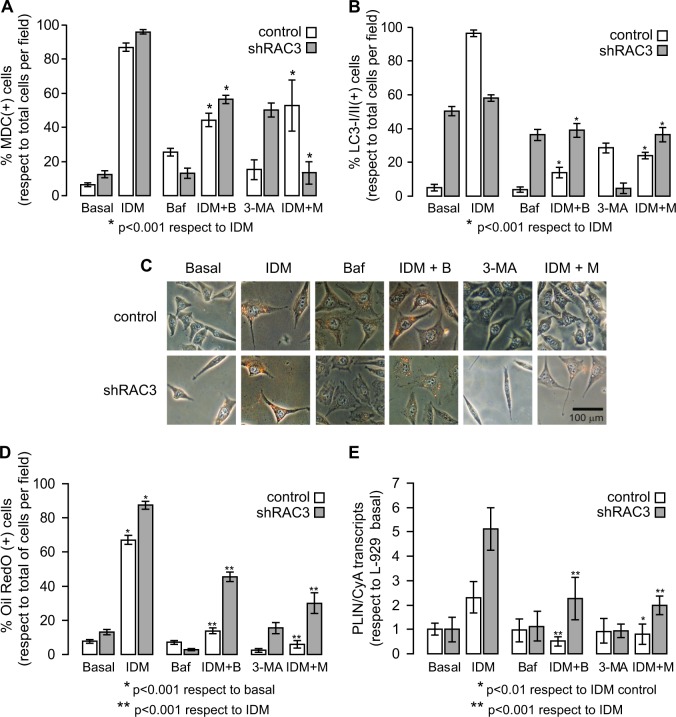
Effect of autophagy inhibitors over adipocyte differentiation. **a**, **b** Control and shRAC3 L-929 cells were seeded in 24-well plates with slices and, after 24 h, cells were treated or not with Bafilomycin A (Baf 5 nM) or 3-Methyladenine (3-MA 0.5 mM) plus IDM. Diagram bars correspond to the percentage of MDC (**a**) or LC3-I/II (**b**) positive cells per field (at least 10 fields per sample). Statistical analysis ANOVA and Tukey post-test *n* = 3 were performed, *p < 0.001 with respect to IDM treatment. **c** Representative images of control and shRAC3 L-929 cells, dyed with Oil Red O, in basal conditions or stimulated with autophagy inhibitors in presence or absence of IDM for 30 h. **d** Diagram bars correspond to the percentage of Oil Red O positive cells per field (at least 10 fields per sample). Statistical analysis ANOVA and Tukey post-test of three independent experiments were performed, *p < 0.001 with respect to basal condition and **p < 0.001 with respect to IDM treatment. **e** PLIN expression in control and shRAC3 L-929 cells at 30 h after treatment with Baf or 3-MA plus IDM. ANOVA (*n* = 3), *p < 0.01 IDM + 3-MA treatment in control cells with respect to IDM treatment and **p < 0.001 IDM plus autophagy inhibitors treatment with respect to IDM treatment

In order to determine the role of autophagy during adipogenesis, we first studied the capacity of two commonly used inhibitors to reverse the effect of IDM on autophagy: Bafilomycin A (Baf) which blocks late autophagy and does not allow the fusion of autophagosomes with lysosomes, and 3-Methyladenine (3-MA) which blocks early autophagy since it is a PI3K III inhibitor. Therefore, control or shRAC3 L-929 cells were stimulated with IDM, in the presence or absence of autophagy inhibitors. Then, we studied autophagy by MDC staining and LC3-I/II labelling. We observed that both inhibitors reverse not only IDM-induced autophagy, but also the basal autophagy due to decrease of RAC3 expression (Fig. 6a, b).

Once the efficiency of these inhibitors had been characterized, the effect of autophagy inhibition on adipocyte differentiation was studied. For this purpose, control or shRAC3 L-929 cell lines were stimulated with IDM in the presence or absence of autophagy inhibitors for 30 h and then, lipid vesicle staining and PLIN expression levels were analysed (Fig. 6c–e). We observed that both inhibitors reduced the percentage of Oil Red O stained positive cells and PLIN expression levels after 30 h of IDM treatment (Fig. 6d, e). This effect was higher in control than in shRAC3 cells.

The weak effect of autophagy inhibitors on adipocyte differentiation in shRAC3 cells cannot be clearly explained with these results. This is probably because the effect of IDM stimulation in cells expressing low levels of RAC3 might involve an additional autophagy-independent way for adipocyte differentiation.

According to all these results, we conclude that the decrease in the coactivator expression contributes to accelerate the differentiation through increasing autophagy, although additional mechanisms could not be excluded.

## Discussion

In this work, we have demonstrated for the first time that RAC3 plays a role in adipocyte differentiation since RAC3 expression is downregulated during this process and, in addition, its constitutive downregulation by a specific shRNA accelerates adipogenesis.

Multiple events contribute to cellular differentiation, in particular, those mechanisms that favour cell cycle arrest and cytoplasm recycling^23,28^. In this regard, we have previously demonstrated that RAC3 is an inhibitor of autophagy, promotes cell proliferation and its expression could be modulated during the cell cycle^18,25^.

Adipogenesis is a complex process that can be divided in two phases: (1) determination and (2) terminal differentiation. Several models have contributed to characterize the events that occur at this last stage, including the pre-adipocyte cell line 3T3-L1^30^. However, the factors involved in the determination phase are less characterized due to the lack of suitable models.

In this work, we have demonstrated that cellular arrest and autophagy, both required for L-929 cells adipogenesis, are increased when RAC3 expression decreases.

To date, several studies have been performed to determine the physiological role of SRC coactivators and, for this purpose, knockout or knockin mouse models have been used^31^. Unlike these studies, in our work we have demonstrated that RAC3 expression levels are downregulated when fibroblasts are differentiated into adipocytes. Moreover, the decrease in RAC3 endogenous expression levels accompanies the appearance of adipocyte markers (positive staining of lipid vesicles and expression of PLIN and PPARγ2).

In 2006, the role of RAC3 has been studied during development of white adipose tissue (WAT)^30,31^. In those studies, the authors have used the pre-adipocyte cell line 3T3-L1 or knockout mice only for RAC3^31^ or in combination with SRC-1^30^, and they have concluded that RAC3 expression is necessary to terminal differentiation of WAT. Although an initial analysis of our results could seem contradictory to those published by these authors, the temporal window and the model studied in this work have differences with respect to the one used in other reports. Thus, we have found that constitutive low RAC3 expression contributes to accelerate adipogenesis. That is in agreement with natural endogenous RAC3 downregulation that occurs during the differentiation process (L-929 cells post IDM treatment, 3T3-L1 cells and adipocytes with respect to L-929 cells basal, NIH/3T3 cells and SVC, respectively). However, we have not investigated the effect of a total blockade of RAC3 expression by gene knockout.

In this work, for the first time, it has been used the murine fibroblastic L-929 cell line as a model to differentiate into adipocytes. These cells are derived from connective subcutaneous, areolar and adipose tissue. Therefore, they are probably well linked to the adipocyte lineage^20,32^. Fibroblasts are a heterogeneous population of stromal cells and their differences are maintained in in vitro cultures, supporting the concept of positional identity^20^.

Certain studies support the concept that RAC3 expression levels should be maintained in order to complete the differentiation process^30,31^. Unlike these reports that were performed on pre-adipocytes until complete terminal differentiation^30,31^, our model involves the transition from fibroblasts to immature adipocytes, just when we observed a decrease in the expression levels of RAC3. Therefore, early downregulation of RAC3 in order to start the differentiation does not exclude its requirement to complete the process.

It is well known that RAC3 has a role in proliferation^24,25,33^ and autophagy^18^. These biological functions depend on their acetyltransferase activity and their ability to interact with different proteins^17,19,25^ by favouring the expression of different genes essential to these events^24,34^.

We have previously found that RAC3 overexpression induces CD1 expression^19^. In addition, we have reported that the increase in the levels of this coactivator induces cell proliferation even in the absence of mitogens^25^. Moreover, other groups have demonstrated that the expression of this coactivator is necessary for G1/S phase transition^24^.

Cellular differentiation requires cell cycle arrest and involves changes in the gene expression pattern, both necessary for the morphological changes associated with cellular remodelling. Here we have showed that the induction of adipocyte differentiation involves endogenous RAC3 downregulation (control cells). Simultaneously, this decrease is accompanied by the decrease in the proliferative rate and CD1 expression, the increase in p21 expression, as well as changes in the expression profile of adipocyte-specific proteins and cellular remodelling. All these features have been also confirmed by the results obtained in cells with low expression of RAC3 (shRAC3).

The importance of autophagy in cytoplasmic remodelling is evident, regarding the morphological changes associated with differentiation^28^. As mentioned before, in previous studies of our laboratory, we have observed that RAC3 overexpression inhibits autophagy induced by different stimuli such as Rapa and hypoxia^18^. In this work we have demonstrated that IDM induces autophagy in a similar manner as Rapa does.

We have observed that autophagy influences the degree of differentiation. In fact, when we inhibited this process at early (3-MA stimulation) or late (Baf treatment) stage, we have found that IDM-induced differentiation was inhibited in control cell line. However, when we analysed what happens in shRAC3 cells, we have found that although autophagy inhibitors exert an important effect on the IDM-induced adipocyte differentiation, this effect was lower than in control cells. This result suggests that the decrease in RAC3 bypasses the effect of the inhibitors. Therefore, considering that this coactivator controls the expression of several genes, additional pathways should not be excluded.

Finally, according to these results, we conclude that early adipocyte differentiation involves RAC3 downregulation, allowing a permissive environment that favours cellular arrest and autophagy, both mechanisms required for adipogenesis.

## Materials and methods

### Cell culture and reagents

The murine fibroblastic cell lines, L-929 (ATCC® CCL-1™), NIH/3T3 (ATCC® CRL-1658™) and 3T3-L1 (ATCC® CL-173™), were purchased from American Type Culture Collection (ATCC® Manassas, VA, USA). NIH/3T3 and 3T3-L1 cell lines were grown in Dulbecco’s modified Eagle’s medium (DMEM) and L-929 cell line in low-glucose DMEM. Every medium was supplemented with 10% fetal bovine serum (FBS) (Gibco Laboratories, Grand Island, NY, USA), penicillin (100 U/ml) and streptomycin (100 mg/ml). Cells were maintained at 37 °C in a humidified atmosphere with 5% CO_2_.

Unless stated, reagents were obtained from Sigma Chemical Co. (St Louis, MO), Thermo Fisher Scientific (Waltham, MA) or Santa Cruz Biotechnology, USA.

### Plasmid construction and transfection

The murine short hairpin RNA for RAC3 (shRAC3) and a control scrambled (control) were prepared using OriGene system following the manufacturer’s protocol. Both constructions were cloned into the BamH1/HindIII cloning sites of the pGFP-V-RS shRNA vector. L-929 cells were transfected using Lipoafectamine 2000 (Invitrogen Corp., USA) according to the manufacturer’s protocol. Three days after transfection, the cells were incubated in selection medium containing 0.5 μg/ml Puromycin (Invitrogen Corp., USA). After 14 days of selection, protein and mRNA expression were analysed by immunoblotting and quantitative Real-Time PCR (qPCR), respectively.

### Proliferation assays

L-929 cells stably transfected with the expression vectors for shRAC3 or control, were plated in 96-well flat bottom plates at a density of 8000 cells per well in 100 μl of medium. After 24 h, medium was replaced by IDM containing: insulin 0.1 μg/ml, dexamethasone 1 μM, the phosphodiesterase inhibitor isobutylmethylxanthine (IBMX) 0.5 mM plus the non-steroidal anti-inflammatory indomethacin 0.1 mM or fresh medium. Cells were fixed at specific time points after medium change and the proliferation was determined by staining with 0.5% crystal violet. Absorbance of surviving stained cells was measured at 570 nm.

For tritiated thymidine uptake assays, cells were incubated with 0.01 μCi of methyl-^3^H-thymidine (3H-Thy, New England Nuclear Life Science, Boston, MA, USA) in presence or absence of IDM and incorporated radioactivity was measured in a liquid scintillation β counter (Packard Instruments). Results are expressed as the mean c.p.m. with respect to control cells from triplicate independent experiments ± SD.

### Oil Red O and Hoechst 33342 staining

Intracellular lipid depositions were demonstrated by staining with Oil Red O. At different time points after medium change wild type, control or shRAC3 L-929 cells were stained with 30% Oil Red O in isopropanol for 60 min. The lipid deposition in differentiated cells was visualized using light microscopy. Results are expressed as the percentage ± SD of Oil Red O positive cells per field.

Hoechst 33342 staining was performed to visualize DNA condensation in basal conditions and after IDM induction.

### Western blot analysis

Western blots were performed as previously described^25^. For experiments where CD1 and PPARγ1/2 expression were determined, control or shRAC3 L-929 cells were grown up to 60% of confluence and the medium was replaced by IDM or fresh medium. At specific time points after medium change, cells were harvested and treated with lysis buffer plus protease inhibitors. Samples were separated on 10% SDS-PAGE and electro-transferred to nitrocellulose membranes.

To validate the knockdown efficiency of shRNA, protein extracts were obtained from different passages after initial selection, samples were separated on 6% SDS-PAGE and membranes were incubated with anti-RAC3 antibody.

In anti-LC3-I/II western blot assays, cells were pre-incubated for 30 min with 10 μg/ml E64D and pepstatin A lysosomal and then incubated with IDM for 90 min.

All membranes were blocked with 5% non fat milk and 0.05% Tween-20 and incubated for 2 h at room temperature or over night at 4 °C in TBS plus 0,05% with anti-PPARγ (recognizes isoform 1 and 2, SC-7196), anti-CD1, anti-RAC3, anti-LC3-I/II, anti-p62 and anti-Tubulin antibodies (Santa Cruz Biotechnology, USA). Subsequently, washed membranes were incubated for 1 h with HRP-conjugated secondary antibodies (VECTOR), developed by chemiluminescence (Santa Cruz Biotechnology, USA).

### Real-time PCR

Total RNA was isolated from control or shRAC3 L-929 cells, NIH/3T3 or 3T3-L1 cells by using the TRIZOL protocol (Invitrogen). Reverse transcription was carried out by using the SuperScript II kit (Invitrogen) according to the manufacturer’s instructions. For gene expression analysis, qPCR was performed by using *mus musculus* sequence-specific primers for:

− RAC3 Fw: 5′-ACATGGTGCATATGAACAGC-3′,

Rv: 5′-GATGTCAGCAGTATTTCTGATCG-3′

− Perilipin Fw: 5′-GTCCCTCAGCTCTCCTGTTA-3′,

Rv: 5′-CTCATCACCACGCTCTGTTG-3′

− p21 Fw: 5′-TATCCAGACATTCAGAGCCAC-3′,

Rv: 5′-AGAGACAACGGCACACTTT-3′

− Cyclophilin A (CyA) Fw: 5′-CCACCGTGTTCTTCGACATC-3′,

Rv: 5′-GCTCGAAAGTTTTCTGCTGT-3′

− Actin Fw: 5′-GCCAACCGTGAAAAGATGAC-3′,

Rv: 5′-ACATGGCTGGGGTGTTGAA-3′

### Autophagy assays

L-929 cells were seeded in 6-well plates on 12 mm glass cover slips at a density of 2.5 × 10^5^ cells/well. After 24 h, the medium was changed by fresh medium or IDM. For immunofluorescence assays the cells were fixed at specific time points with 3% formaldehyde and 0.02% glutharaldehyde for 15 min. Incubation with primary antibody against LC3-I/II (Santa Cruz Biotechnology, USA) was performed for 1 h at room temperature in PBS with 10% SFB. Then, the cells were washed with PBS, incubated with a FICT-labelled secondary antibody for 1 h, washed with PBS and mounted over glass cover slips with PBS/Glycerol 1:1 solution.

Autophagy induction was monitored by MDC incubation. The percentage of positive cells (showing granular staining) was determined by counting a minimum of 100 cells per slide using fluorescence microscopy.

Rapamycin (0.5 μM), a potent autophagy inductor, was used as control. In order to perform the assays with autophagy inhibitors, cells were stimulated with Bafilomycin A (Baf 5 nM) or 3-Methyladenine (3-MA 0.5 mM) for 30 min before IDM treatment.

In all cases, the cells were analysed with an Olympus BX51 fluorescent microscope. Images were taken with a digital camera and analysed with NIH-ImageJ software.

### Analysis of Gene Expression Omnibus (GEO)

To compare the RAC3 expression levels between stromal vascular cells and adipocytes from murine epididymal adipose tissue, we used values obtained from GSE65557 data bank, platform GPL6246 Affymetrix (Santa Clara, CA, USA)^22^.

### Statistical analysis

At least three independent experiments were carried out for all assays. Results were expressed as the mean ± SD. The significance of differences between experimental conditions was determined using Student test for paired observations or ANOVA and the Tukey Multiple Comparisons Test for paired observations.
